# Non-Mendelian transmission of X chromosomes: mechanisms and impact on sex ratios and population dynamics in different breeding systems

**DOI:** 10.1042/BST20231411

**Published:** 2024-08-16

**Authors:** Sally Adams, Andre Pires-daSilva

**Affiliations:** School of Life Sciences, University of Warwick, Coventry CV4 7AL, U.K.

**Keywords:** evolution, meiotic drive, sex determination, sex ratio

## Abstract

The non-Mendelian transmission of sex chromosomes during gametogenesis carries significant implications, influencing sex ratios and shaping evolutionary dynamics. Here we focus on known mechanisms that drive non-Mendelian inheritance of X chromosomes during spermatogenesis and their impact on population dynamics in species with different breeding systems. In *Drosophila* and mice, X-linked drivers targeting Y-bearing sperm for elimination or limiting their fitness, tend to confer unfavourable effects, prompting the evolution of suppressors to mitigate their impact. This leads to a complex ongoing evolutionary arms race to maintain an equal balance of males and females. However, in certain insects and nematodes with XX/X0 sex determination, the preferential production of X-bearing sperm through atypical meiosis yields wild-type populations with highly skewed sex ratios, suggesting non-Mendelian transmission of the X may offer selective advantages in these species. Indeed, models suggest X-meiotic drivers could bolster population size and persistence under certain conditions, challenging the conventional view of their detrimental effects. Furthering our understanding of the diverse mechanisms and evolutionary consequences of non-Mendelian transmission of X chromosomes will provide insights into genetic inheritance, sex determination, and population dynamics, with implications for fundamental research and practical applications.

## Introduction

Non-Mendelian inheritance describes patterns of genetic transmission that deviate from the principles established by Gregor Mendel [1]. In contrast with Mendelian inheritance, where alleles segregate independently with equal probability, non-Mendelian mechanisms skew these probabilities. A key example of non-Mendelian inheritance is meiotic drive [2]. Meiotic drivers are selfish DNA elements that manipulate the meiotic process to increase their transmission to offspring, while typically having detrimental effects on the rest of the genome. This leads to the evolution of suppressors to counteract their negative effects creating an ongoing evolutionary arms race. When meiotic drive affects sex chromosomes, it can lead to significant distortion in sex ratios, influencing the population dynamics and evolution of species [3].

**Figure 1.**
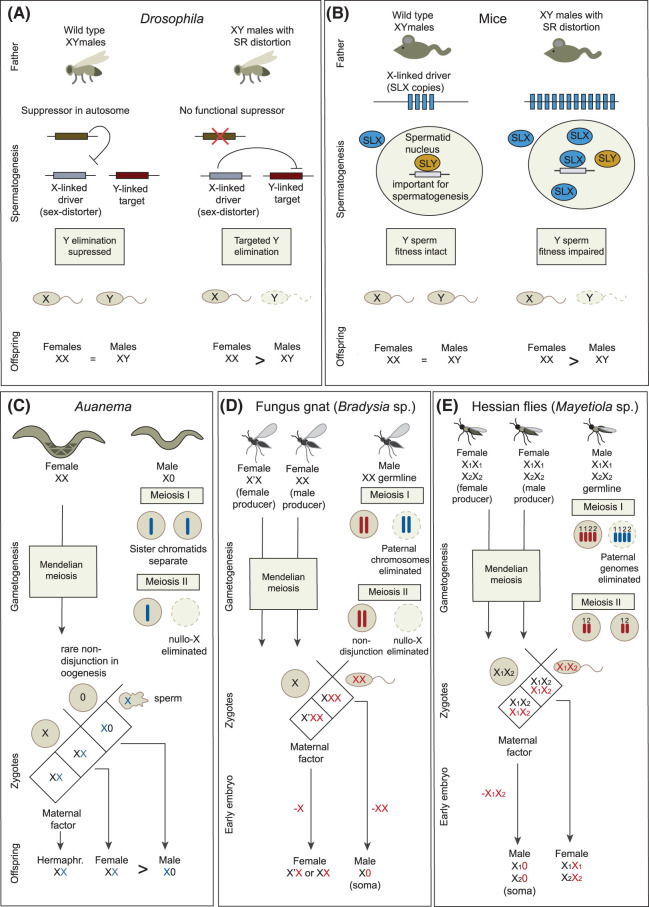
Non-Mendelian inheritance of the X chromosome during spermatogenesis. (**A**) In wild-type male *Drosophila*, inhibition of an X-linked driver by an autosomal suppressor ensures equal production of X-bearing and Y-bearing sperm and balanced sex ratios. In sex ratio (SR) distorter males, the autosomal repressor activity is absent. The X-linked driver selectively eliminates the Y-target driving preferential production of X-bearing sperm and female-skewed populations. (**B**) In wild-type male mice, X-linked SLX and Y-linked SLY repeats, which encode antagonistic transcriptional regulators, have balanced copy numbers. In the nucleus, SLY repression complexes outcompete SLX activation complexes, ensuring equal fitness of X- and Y- bearing sperm. If the ratio of SLX to SLY repeats increases, SLX complexes outcompete SLY, leading gene expression patterns which negatively impact the fitness of Y-bearing sperm, resulting in female-skewed populations. (**C**) *Auanema* XX females produce mostly (∼90%) haplo-X oocytes. A small number (∼10%) of nullo-X oocytes are produced by non-disjunction. *Auanema* X0 males exhibit non-Mendelian X chromosome inheritance during spermatogenesis. In meiosis I, the sister chromatids of the X are separated into different daughter cells. In meiosis II, haplo-X cells develop into functional spermatids. The production of only X-bearing sperm results in populations with very few males. Maternal factors deposited in the oocyte are thought to regulate XX zygote sexual differentiation into either the female or hermaphrodite adult. (**D**) *B. coprophila* males have an XX germline genome (and an X0 somatic genome). In meiosis I, all paternal genomes, including the X (shown in blue), are eliminated leaving only maternally inherited genomes (in red). In meiosis II, the X chromatids fail to separate, co-segregating to the same daughter cell to form diplo-X sperm. *B. coprophila* females produce haplo-X oocytes, so all zygotes are triploid-X. In zygotes from gynogenic mothers (X′X) maternal factors are thought to drive somatic elimination of one paternal X to give female broods, while zygotes from androgenic mothers (XX) eliminate both paternal X chromosomes to give male broods. (**E**) *M. destructor* has two X chromosomes (X_1_ and X_2_). Males have an X_1_X_1_X_2_X_2_ germline genome (X_1_X_2_00 soma). In meiosis I, all paternal genomes are eliminated, resulting in haploid X_1_X_2_ sperm. Paternal genome elimination during somatic development determines sex. In embryos from gynogenic mothers, maternal factors inhibit paternal genome elimination to give female broods. Embryos from androgenic mothers eliminate the paternal genome to give male broods. Diagrammatically, paternally inherited X chromosomes and chromatids are shown in blue, and maternally inherited X chromosomes and chromatids are shown in red.

Research on non-Mendelian sex chromosome transmission has primarily focused on X-meiotic drive in the XX/XY systems of mice and *Drosophila*. In these systems, the suppression of X-meiotic drive is the evolutionarily stable state, ensuring the equal production of male and female offspring (Figure 1A,B) [4,5]. However, in some less well-known insect and nematode species with XX/X0 sex determination the opposite is true: unsuppressed X-meiotic drive and skewed sex ratios appear to be the evolutionary stable state (Figure 1C–E) [6–14]. This suggests that, contrary to current thinking, non-Mendelian X chromosome transmission can offer a selective advantage in certain species and breeding systems.

As large-scale genome sequencing projects, such as the Darwin Tree of Life Project [15], generate chromosomal-level genome assemblies from all walks of life, it is likely that numerous further examples of natural unsuppressed X-meiotic drive will be uncovered. This will have significant implications, from expanding on our understanding of the regulation of non-Mendelian transmission to the development of conservation strategies. Furthermore, current models of X-meiotic drive are extremely complex, largely due to the ongoing intragenomic conflict between meiotic drivers and suppressors, making it challenging to unravel the mechanisms that drive and supress non-Mendelian X transmission [4,5]. Species where active X-meiotic drive is the evolutionary stable state could offer simpler models for identifying key conserved genetic factors driving non-Mendelian X chromosome transmission.

Mechanistically, non-Mendelian X chromosome inheritance may occur via unequal transmission of the X during oogenesis or spermatogenesis [16]. There are currently only a few examples of non-Mendelian X transmission during oogenesis, primarily focusing on the non-random segregation of the X in aneuploidy mutants of mice (X0) [17,18] and *Caenorhabditis elegans* (XXX) [19,20]. Wild-type populations of the free-living nematode *Auanema rhodense* exhibit non-Mendelian transmission of the X during oogenesis. In this species, XX hermaphrodites actively exclude the X to produce nullo-X oocytes [21]. However, as the hermaphrodites also produce diploid-sperm, self-fertilisation results in the XX progeny predicted by Mendel's laws, albeit by an unusual route.

Here we compare the known regulatory mechanisms and impact on sex ratios of non-Mendelian X chromosome transmission during spermatogenesis in systems where the X-meiotic drive is either supressed or unsuppressed in wild-type populations. Furthermore, we examine the potential costs and benefits of the X-linked meiotic drivers in these different systems and the evolutionary implications.

## Suppression of non-Mendelian X chromosome transmission

Nearly a century ago, Gershenson documented sex ratio distortion (SR) in *Drosophila obscura* [22], noting that certain males tended to produce mostly female offspring. He attributed this phenomenon to a factor on the X chromosome, which prevented the ability of Y-carrying sperm to fertilise oocytes. Indeed, sex chromosome meiotic drive in males includes at least two components: a driver linked to the sex chromosome and a target.

In wild-type *Drosophila* the activity of the X-linked driver is inhibited by a suppressor (Figure 1A). In SR males, suppression is lifted, and the X-linked driver facilitates its transmission by selectively eliminating gametes carrying the Y-target [4]. Drivers employ various methods to induce the production of non-viable Y-bearing spermatids, including Y-chromosome non-disjunction in the Paris SR system of *Drosophila simulans* [16,23], Y-chromosome degeneration during anaphase II in *Drosophila pseudoobscura* and *Drosophila athabasca* [24], and defective DNA condensation and differentiation during sperm maturation in the Winters SR system of *D. simulans* [23] (Figure 1A).

Most drivers in *Drosophila* arise from gene duplication events [4]. Gene duplication serves as a powerful mechanism for genetic innovation, enabling the evolution of novel functions in the new gene copy while preserving the original function of the ancestral gene [25,26]. In *Drosophila*, the mechanisms for X-linked drivers have been associated with heterochromatin formation, small RNA pathways, and nuclear transport. For example, in the *D. simulans* Paris SR system, the heterochromatin protein 1 D2 (HP1D2), derived from a duplication of HP1D, plays a critical role in the X chromosome drive [27]. Specifically expressed in male premeiotic germ cells, HP1D2 selectively binds to the Y chromosome to ensure that sister chromatids separate correctly during meiosis. If HP1D2 is non-functional, it results in non-viable spermatids. As a result, only X-bearing spermatids remain viable. In *Drosophila neotestacea*, a testis-specific X-linked duplicate of the nuclear transport gene *importin-α2* is overexpressed in males with sex ratio distortion. This gene is a leading candidate for being an X-linked driver [28].

In mice, multiparent mouse mapping populations, where multiple founder strains are mated to disrupt the original haplotypes, have been used to identify loci that defy Mendel's laws [29]. Analysis of breeding records from the Collaborative Cross lines [30] found over a third showed significant sex ratio distortion [31]. The authors speculated that the shuffling of eight diverse parental genomes resulted in decoupling sex ratio distorters and their co-evolved suppressors, releasing complex multiallelic systems of sex chromosome drive.

One of the best-characterised sex-linked driver/suppressor systems in mice is the Sycp3-like gene family (Figure 1B) [5,29,32–34]. This gene family codes for antagonistic transcriptional regulators, with 50–100 copies located on the X (*Slx/Slxl1* (*Sycp3-like X-linked* and *Slx-like 1*)) and Y (*Sycp3-like Y-linked*) chromosomes [35,36]. Although these genes are only expressed in haploid spermatids, they share cytoplasm as they develop in pseudosyncytium [37]. Thus, SLX and SLY proteins are found in both X- and Y-bearing spermatids [5]. In wild-type mice, with balanced copy number ratios of *Slx* and *Sly* loci, the SLY protein outcompetes SLX (Figure 1B). However, if there is an excess of X-linked copies, SLX protein can outcompete the SLY repression complex, leading to impaired fitness of Y-bearing sperm, reduced fertility, and female-biased litters [38,39]. Conversely, the knockdown of *Slx* expression results in several meiotic defects and male-biased litters [38]. The mechanisms by which such imbalances lead to sex ratio distortion are not yet fully understood, not least due to the structural complexity of the regions and the large number of genes targeted by SLX and SLY [5].

In mice and *Drosophila*, the evolutionarily stable state occurs when the drivers and suppressors are balanced (Figure 1A,B). However, in some species with an XX/X0 sex-determination system, active X-meiotic drive and skewed sex ratios appear to be the evolutionarily stable state (Figure 1C–E). Males of the nematodes *Auanema* and *Strongyloides*, as well as fungus gnats and gall midges, are X0 animals. However, due to unequal allele segregation during spermatogenesis or the selective elimination of non-X bearing spermatocytes, produce only X (or XX) bearing sperm [6–14]. Strikingly, in many cases maternally inherited factors then appear to regulate sexual development in the offspring, either by modulating passage through larval diapause in *Auanema* or by inducing paternal X chromosome elimination in the early embryo in fungus gnats and gall midges.

## Non-Mendelian X chromosome transmission in wild-type populations

In the rat parasitic nematode *Strongyloides ratti*, selection for X-bearing sperm occurs post-meiotically. Inheritance of the X during male spermatogenesis occurs as expected for an X0 individual [6]. Half of the spermatocytes inherit an X, while half do not. However, nullo-X spermatocytes appear to undergo apoptosis, so that all viable mature sperm carry an X chromosome. In contrast, in the free-living nematode genus *Auanema*, the drive to retain only X-bearing sperm occurs via a modified meiosis.

*Auanema* nematodes are trioecious: outcrossing females and males coexist in the same population as self-fertilising hermaphrodites (Figure 1C). Animals with one X chromosome differentiate into males, whereas animals with two X chromosomes undergo non-male development [7,40]. Maternal age or environment then determines non-male fate [41,42].

*Auanema* males (X0) and hermaphrodites (XX) exhibit non-Mendelian segregation of the X during spermatogenesis. In meiosis I, the sister chromatids of the univalent X in males, or the X homologs in hermaphrodites, are separated prematurely and inherited by different daughter cells [7,8] (Figure 1C). In meiosis II, the lone X chromatid in males or both X chromatids in hermaphrodites segregate together with essential sperm components into one daughter cell, which subsequently develops into the haplo-X spermatid in males or the diplo-X spermatid in hermaphrodites. The remaining DNA is discarded in the non-X-bearing daughter cell and is eliminated via apoptosis [7,8]. Since *A. rhodense* males exclusively produce sperm carrying the X chromosome, the progeny of male-female crosses will be male only if the X-bearing sperm fertilise nullo-X oocytes, which are believed to arise from X non-disjunction during female oogenesis [21]. Recent QTL analysis of recombinant inbred lines in another *Auanema* species, *Auanema freiburgense*, suggests that this non-Mendelian segregation of the X during spermatogenesis is governed by epistatic interactions between a region on the X chromosome and autosomal factors [43].

In fungus gnats, both meiotic divisions of spermatogenesis exhibit non-Mendelian traits. During meiosis I, all maternally inherited chromosomes are retained, while all paternally inherited chromosomes are excluded in a bud and discarded (Figure 1D) (reviewed in [11]). In meiosis II, a cis-acting regulatory element on the X chromosome inhibits centromere function, and no kinetochore forms [9,10]. This leads to non-disjunction of the X so both X chromatids are inherited by the same daughter cell, which then develops into the functional sperm. The non-X-bearing daughter cell is eliminated [9]. Consequently, every sperm exclusively carries only maternally inherited chromosomes and is diploid for the (maternally inherited) X chromosome.

The hessian fly (*Mayetiola destructor*) also exhibits non-Mendelian removal of the paternally inherited chromosomes (Figure 1E). Strikingly, it has two non-homologous X chromosomes, X_1_ and X_2_ [12]. During meiosis I, all paternally inherited chromosomes are eliminated, resulting in haploid sperm with only maternally derived X_1_X_2_ chromosomes [12–14].

Maternally inherited factors also regulate sexual fate. In *Auanema*, maternal age [41] or environment [42,44] determines if XX progeny develop into females or self-fertilising hermaphrodites by regulating passage through larval diapause, probably via maternally inherited factors (Figure 1C). In fungus gnat and gall midges, maternally supplied factors regulate sex by controlling the elimination or retention of paternal chromosomes [45,46]. Fungus gnat zygotes start as XXX individuals and either one or two paternally derived X chromosomes are lost from somatic cells during early embryogenesis, driving female (XX) or male (X0) development (Figure 1D). The chromosomes to be eliminated do not divide during anaphase and remain on the metaphase plate [47]. Many well-studied fungus gnat species are monogenic, whereby individual mothers produce only female (gynogenic) or male (androgenic) offspring. In *Bradysia coprophila* and *B. impatiens* monogeny is associated with X-linked chromosomal inversions [48–50], and the affected chromosome is termed X prime (X′). Gynogenic mothers are X′X and produce embryos that eliminate one paternal X chromosome, while androgenic mothers are XX and produce embryos that eliminate both paternal X chromosomes [51]. The process also uses the Controlling Element, which likely acts as a recognition site for X elimination [46,49,50]. X′ is thought to encode maternal factors that regulate the loss of only one paternal X.

Chromosomal inversions also regulate sex determination via X elimination during *M. destructor* embryonic development. In this species, gynogenic mothers are heterozygous for a large autosomal inversion which is presumed to contain regions that repress X elimination (Figure 1E) [12,14,51]. For example, in androgenic mothers, which do not carry the inversion, paternally inherited X_1_X_2_ are eliminated in the early embryo, presumably mediated by the encoded maternally provided factors, to produce male-only broods.

## Costs and benefits of X-linked meiotic drivers in species with different breeding systems

In most well-studied systems, X-linked meiotic drivers are associated with fitness costs by reducing the viability or fecundity of carriers [52]. X meiotic drive in males leads to female-biased broods, which could result in population extinction if insufficient males mate with the females [53]. This can be negated by the suppression systems discussed above, or by direct fitness costs associated with the driving allele [54–56]. Selection likely favours chromosomal inversions that maintain the genetic linkage between drivers and their suppressors. However, these inversions can also capture deleterious mutations or allow them to accumulate through the lack of recombination (Muller's ratchet). For example, in the SR system of *Drosophila recens* the X-chromosome contains multiple inversions that likely prevent recombination between the SR and wild-type X chromosomes creating a large haplotype of 130 cM [57]. SR homozygous females are sterile, while heterozygous females and males carrying one SR-X chromosome exhibit reduced fertility (males via sperm competition) [57,58]. These apparent fitness costs keep SR chromosome occurrence low in natural populations [58].

However, in other species X-meiotic drive-like systems appear stably unsuppressed in wild-type populations, suggesting they confer a selective advantage and not a fitness cost (Figure 1C–E). For example, in *Auanema*, wild-type populations exhibit stable X-meiotic drive during male spermatogenesis, resulting in few male offspring. However, when the genomes of two of these low male-producing strains were shuffled, by crossing them to create recombinant inbred lines, half of the lines exhibited Mendelian X-chromosome inheritance and non-skewed sex ratios [43]. This loss of X-meiotic drive presumably occurs by decoupling strain-specific X-linked drivers and genetic elements that promote the activity of those drivers.

Mathematical models have shown that X-meiotic drivers can theoretically boost population size, especially when only a few males are required to fertilise a female-biased population [59]. This effect of X-meiotic drivers may be underestimated due to their well-documented detrimental effects in model systems, such as mice and *Drosophila*. Elucidating the role of unsuppressed X-meiotic drivers in wild-type species with different breeding systems may uncover conserved cellular mechanisms that regulate non-Mendelian chromosomal inheritance. Furthermore, X-meiotic drivers may be used as a method of biocontrol, and it is therefore crucial to understand the potential selective advantage of such drivers in different populations [60]. In simulations using an engineered X-meiotic driver that selectively eliminates the Y chromosome in male mice, effectively converting XY males into X0 females, population control was influenced by two main factors: the efficiency of the X-meiotic driver and the mating system of the targeted species. When the X-driver was highly effective at removing the Y chromosome, it significantly reduced the male population. This, in turn, restricted the number of females that could reproduce in each cycle, ultimately causing a population crash. However, in scenarios where the X-meiotic driver was less efficient and males engaged in polygynous breeding (mating with multiple females), the resultant sex ratio bias towards females, coupled with an adequate number of males, led to an increase in population size [60].

The study of non-Mendelian inheritance of genetic sex determination has important implications for fundamental principles in genetics and evolution, as well as practical applications like pest management. For instance, the selective elimination of paternal chromosomes in *Bradysia* [49] advanced our understanding of genomic imprinting, which is also crucial for mammalian development. Additionally, the formation of viable X-spermatid and nullo-X spermatid in *Auanema* provides insights into the mechanisms of asymmetric cell divisions [7,8,43]. Understanding the evolution of meiotic drivers may reveal strategies to engineer them to establish new pest control strategies, potentially improving the management of insect populations sustainably.

## Perspectives

- The study of non-Mendelian inheritance of sex chromosomes is crucial for understanding the driving forces that influence sex ratios in populations, which has implications for evolution and pest management.
- Current research focuses on mechanisms of meiotic drive that influence sex chromosome inheritance and how these can lead to skewed sex ratios.
- Future research will explore the molecular mechanisms behind non-Mendelian transmission patterns and their evolutionary advantages or disadvantages. Insights from this field could lead to innovative approaches in pest control, conservation strategies, and understanding human genetic diseases related to sex chromosome anomalies.

## Abbreviations

HP1D2: heterochromatin protein 1 D2
SR: sex ratio
